# Semi-Empirical Estimation of Dean Flow Velocity in Curved Microchannels

**DOI:** 10.1038/s41598-017-13090-z

**Published:** 2017-10-20

**Authors:** Pouriya Bayat, Pouya Rezai

**Affiliations:** Department of Mechanical Engineering, York University, Toronto, ON Canada

## Abstract

Curved and spiral microfluidic channels are widely used in particle and cell sorting applications. However, the average Dean velocity of secondary vortices which is an important design parameter in these devices cannot be estimated precisely with the current knowledge in the field. In this paper, we used co-flows of dyed liquids in curved microchannels with different radii of curvatures and monitored the lateral displacement of fluids using optical microscopy. A quantitative *Switching Index* parameter was then introduced to calculate the average Dean velocity in these channels. Additionally, we developed a validated numerical model to expand our investigations to elucidating the effects of channel hydraulic diameter, width, and height as well as fluid kinematic viscosity on Dean velocity. Accordingly, a non-dimensional comprehensive correlation was developed based on our numerical model and validated against experimental results. The proposed correlation can be used extensively for the design of curved microchannels for manipulation of fluids, particles, and biological substances in spiral microfluidic devices.

## Introduction

Curved and spiral microchannels have a wide range of applications in microfluidic devices^1,2^ such as isolation of circulating tumor cells^3^, cell enrichment^4^, bacteria separation^5^, fluid mixing^6,7^, microfilteration^8^, and inertial focusing and separation of particles^9–15^. Fluids flowing in the axial direction of a curved channel experience a pressure gradient along the radial direction^14^. This leads to continuous circulation of the fluid in the radial direction and subsequent formation of two or more counter-rotating vortices in the channel^16,17^. This lateral recirculating flow is called a secondary (or Dean) flow^18–20^. Formation of Dean flow in curved and spiral channels is inevitable, yet their presence may be dominant and useful in some applications^21–23^ while undesirable in others^24,25^.

Understanding the causes and effects of Dean flow in curved channels is of paramount importance to design of microfluidic devices. For instance, it has been reported that secondary flows can skew the velocity profile in arterial bends and affect the shear distribution in blood vessels^26^. This influences low density lipoproteins (LDL) transport through the vascular network and enhances the risk of atherosclerosis^27,28^. As an additional example, Martel and Toner^29^ reported the necessity of existence of an accurate formula to predict the velocity at which particle focusing occurs in a spiral channel. They took two different approaches to investigate the inertial focusing, one by ignoring and the other by including the Dean velocity (V_De_). Using the former approach, they could only determine the width required for particle focusing. However, by numerical simulation of Dean flow in their channel, they could calculate the average V_De_ required for the later approach to find both the width and axial velocity required for inertial focusing. Accordingly, predicting the Dean flow characteristics in curved microchannels, and specifically the average Dean flow velocity, with respect to fluid properties (e.g., density and viscosity) and channel specifications (e.g., width, height and radius of curvature) has been the center of attention of a few research articles^10,30^.

Attempts to characterize the Dean flow in curved microchannels can be categorized to the use of analytical, numerical and experimental approaches. Two dimensionless numbers, namely the Reynolds (Eq. 1) and the Dean (Eq. 2) numbers, are used widely to describe the primary and secondary fluid flow behaviors in a channel.

1 $${Re}=\frac{\rho {V}_{x}D}{\mu }$$

2 $$De={Re}\sqrt{\frac{D}{2R}}$$

where ρ [kg m^−3^] and *μ* [kg m^−1^s^−1^] denote the fluid density and dynamic viscosity, respectively. Geometrical properties of the channel are represented by *D* [m] and *R* [m] which are the hydraulic diameter and radius of curvature of the channel. Lastly, *V*_*x*_ [m s^−1^] is a characteristic velocity that is often taken to be the average axial velocity of the fluid. Reynolds number presents the ratio of inertial forces to viscous forces and is a representative of the fluid flow regime. Fluid flow in curved channels is highly influenced by the lateral acceleration of the fluid due to centripetal forces. Dean number indicates the ratio of the inertial and centripetal forces to the viscous forces^31^ and is widely used to characterize the strength of secondary flows in curved channels^32^. Dean number commonly alters in the range of 0–30 in microfluidic devices^11,20^.

The exact analytical solution of Navier-Stokes equations for fluid flow in curved microchannels does not still exist but efforts have been made to simplify these highly nonlinear coupled equations and solve them using mathematical approaches such as perturbation and spectral methods. Norouzi and Biglari^33^ used curvature ratio as the perturbation parameter to solve the secondary flow problem in curved ducts. Although their solution showed acceptable agreement with experimental results but associated errors were remarkably high for sharp curvatures that are prevalent in microfluidic devices. Dual solutions for circular curved tubes were proposed by Yanase *et al*.^34^ but the high Dean number in their solution (order of 1000) is not directly applicable to microfluidic applications in which the Dean number is relatively low (De < 30). Analytical solutions for elliptical^35^ cross sections and non-Newtonian fluids such as Bingham Plastics^36^ and second order fluids^37^ have also been reported using the perturbation method. However, extensive simplifications in analytical solutions limit their application for real life problems and make them not suitable for investigation of secondary flows in curved microchannels especially if multi-phase flows (e.g., particles in water) are to be investigated. Therefore, analytical methods have not been successful yet to offer a reliable solution that can conveniently be used for determination of Dean flow velocity at low Dean numbers in microfluidic devices.

Zhang *et al*.^38^ took a numerical approach to investigate the effects of Coriolis and centrifugal forces on flow patterns (De > 100) and the friction factor in square and rectangular channels with aspect ratios of lower and higher than one. Their observations led to the conclusion that four types of vortices can co-exist in the channels due to the effects of centrifugal and Coriolis forces and their associated instabilities. Ookawara *et al*.^30^ numerically studied Dean flows in a rectangular curved microchannel and proposed two correlations, both in the form of power functions, for estimation of average (Eq. 3) and maximum Dean flow velocities.

3 $${V}_{De}=1.8\times {10}^{-4}\,D{e}^{1.63}\,\frac{m}{s}$$

Although the correlations proposed by Ookawara *et al*.^30^ have been widely adopted for design of spiral microchannels, the effects of many influential parameters such as fluid viscosity and channel hydraulic diameter, aspect ratio, and radius of curvature were not investigated in-depth. Altogether, there is a need for development and experimental validation of numerical models that can provide a more accurate estimation of the average Dean flow velocity in spiral and curved microchannels.

At the experimental level, Ligrani and Niver^39^ studied the effect of Dean number (40 < De < 220) in a curved channel with large aspect ratio (width/height) of 40 and reported formation of counter-rotating vortices for Dean numbers greater than 64. A set of experiments in a square curved channel were performed by Bara *et al*.,^40^ and their results revealed formation of two or four symmetric vortices at De = 125 and De = 137, respectively, demonstrating the importance of channel dimensions and fluid properties in formation of secondary flows. Yamamoto *et al*.^41^ experimentally visualized secondary flow vortices in a curved channel with rotating and stationary walls. They characterized the shape of Dean flow vortices for different Taylor (Tr = $\frac{{w}^{2}{\rm{\Omega }}}{\sqrt{2\delta }\nu }$, where $w$ is the width of the channel, and ${\rm{\Omega }},\,\delta \,$and $\nu$ are angular velocity, non-dimensional curvature and kinematic viscosity, respectively) and Dean numbers in a square cross section channel and provided a flow pattern diagram showing the layout and number of vortices on various Tr-De diagrams. Dispersed microparticles can also be used for flow visualization and velocity field determination^42^, however, it has been already demonstrated that particles are greatly affected by inertial forces^43^ in straight and spiral microfluidic devices and specially when the size of the channel is comparable to the size of the microparticles. Thus, particles recirculation velocity may not be the best representative of Dean flow velocity in a curved microchannel. There is a need for development of more reliable experimental approaches for parametric investigation of the average Dean velocity in spiral and curved microfluidic devices.

In this paper, we have investigated the effect of various deterministic parameters on Dean flow velocity in curved microchannels using simple but practical experimental and numerical approaches. The studied parameters can be categorized into geometrical characteristics (e.g. channel width, height, hydraulic diameter, and radius of curvature) and fluidic properties (e.g. axial velocity and kinematic viscosity). With this comprehensive investigation, we were able to propose a non-dimensionalized correlation for estimation of V_De_ in curved microchannels (at De < 30) that can be used widely for design of curved and spiral microfluidic devices.

## Results and Discussions

**Figure 1:**
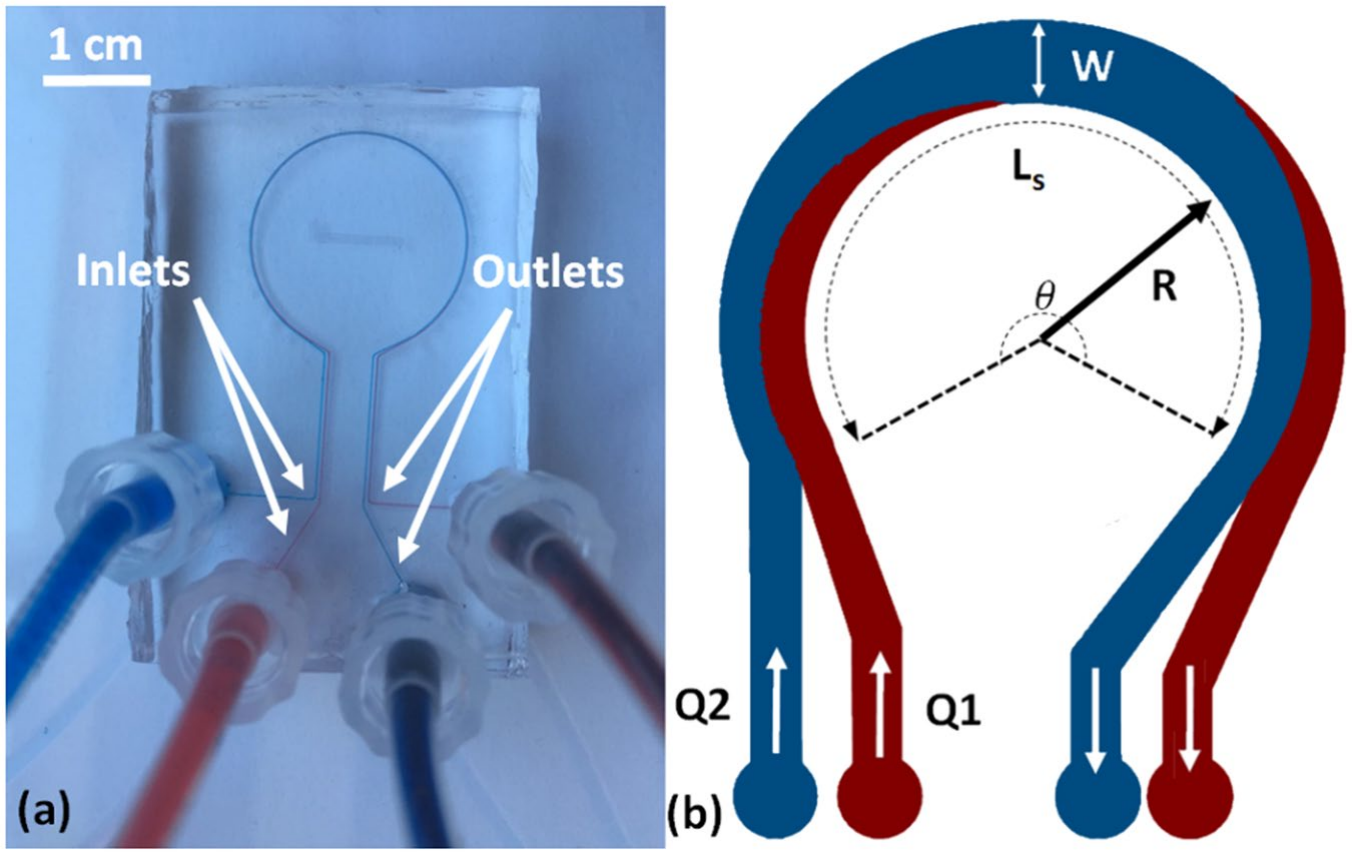
A microfluidic device consisting of a curved microchannel with a radius of curvature of R = 1 cm and cross section area of w = 300 *μ*m by h = 150 *μ*m that was used for experimental investigation of the average Dean velcosity (V_De_). (**a**) Water streams dyed with methylene blue and red food dye were introduced from the two inlets and their radial displacement was imaged. This is demonstrated schematically in (**b**). Microchannels with four radii of curvatures (R = 0.5, 1, 1.5 and 2 cm all with 150 *μ*m × 150 *μ*m cross-section) and two additional cross-sectional dimensions (100 *μ*m × 150 *μ*m and 300 *μ*m × 150 *μ*m both with R = 1 cm) were used in our studies.

The microfluidic devices used in this study were made of polydimethylsiloxane (PDMS) and consisted of a curved microchannel with $\theta$ = 330°, two inlets and two outlets as shown in Fig. 1a. The effect of radius of curvature (R = 0.5, 1, 1.5 and 2 cm) on fluid recirculation (schematically shown in Fig. 1b) and Dean flow velocity (V_De_) was investigated in square cross-section (150 µm × 150 µm) curved microchannels. Two additional devices with R = 1 cm and rectangular cross-sections of 100 µm × 150 µm and 300 µm × 150 µm were used to investigate the effect of channel dimensions (width, height and hydraulic diameter) on Dean flow velocity. Two additional properties studied were fluid axial velocity (V_x_) and kinematic viscosity as fully discussed in the following sections.

### Quantification of Dean Flow in Curved-Channel Microfluidic Devices using a Switching Index (SI)

**Figure 2:**
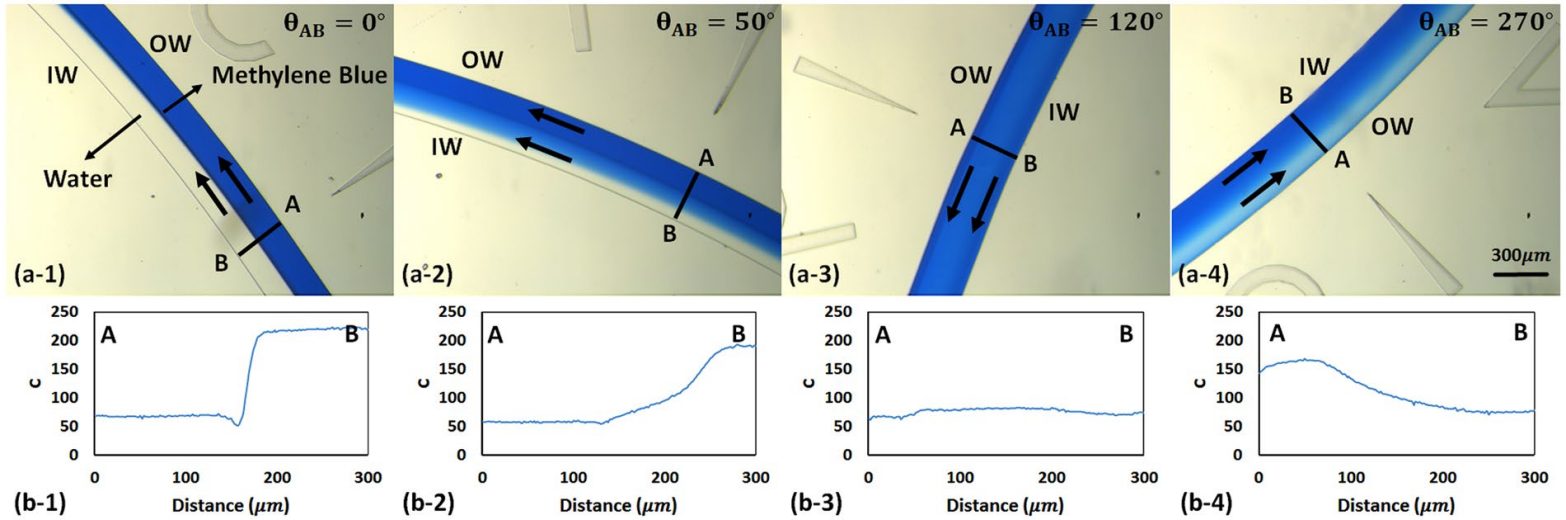
Co-flow images of methylene blue (MB) and water at inlet flow rate of 0.6 mL min^−1^ (i.e., velocity of 0.22 m s^−1^) along the length of a 300 µm × 150 µm curved microchannel with R = 1cm (**a-1** to **a-4**) and intensity diagrams corresponding to assessment lines AB along the width of the channel at specific control points in each image (**b-1** to **b-4**). (**a-1** and **b-1**) show the beginning of the channel where the intensity diagram was similar to a step function and the fluids were completely separated, i.e., water at Inner Wall (IW) and MB at Outer Wall (OW). In (**a-2** and **b-2**), MB and water started to displace laterally due to Dean flow vortices. In (**a-3** and **b-3**)_,_ water was completely sandwiched in between MB layers, and the intensity diagram was almost uniform. In (**a-4** and **b-4**), water and MB started to appear closer to the OW and IW, respectively, hence demonstrating switching in position compared to their initial conditions.

We first focused on developing a simple and accurate experimental technique to quantify average Dean velocity in our curved-channel microfluidic devices. For this, methylene blue (MB) and water solutions were co-flown into the devices at different axial velocities and videos of fluid recirculation were recorded along the channel under a microscope (see Supplementary Videos 1 and 2). The videos were then imported into the freeware ImageJ (National Institutes of Health, Bethesda, Maryland, USA) for analysis of target cross sections along the curved microchannels. For instance, Fig. 2a shows the snapshots of the R = 1cm device along the channel with a co-flow of water and MB at a flow rate of 0.6 mL min^−1^ (V_x_ = 0.22 m s^−1^).

Intensity values (*c*_*i*_) across assessment lines (e.g. line AB in Fig. 2a) drawn at 10 degree intervals along the channel were obtained (Fig. 2b) and an average intensity ($\bar{c}$) for each analyzed line was calculated from the obtained cross-sectional intensity values. To obtain a quantitative assessment of the secondary Dean flow and fluid recirculation, the intensity values obtained in Fig. 2b were used in Equation (4) to calculate the $\sigma$-values (standard deviation of intensity values) at each assessment line AB along the channel. Subsequently, a normalized index called the *Switching Index* ($SI$) was derived for each assessment line using Equation (5) and plotted along the channel for quantification of Dean flow characteristics such as Dean velocity. The *SI* was defined based on an index originally used to characterize mixing in microfluidic devices^44^. Although diffusive mixing was not significant in our device (Peclet number ~10^5^) due to the high axial flow velocities. The *SI* trends properly represented the lateral displacement of fluids due to Dean flow in our curved microchannels.

4 $$\sigma =\sqrt{\frac{1}{N}\sum _{i=1}^{N}{({c}_{i}-\bar{c})}^{2}}$$

5 $$SI=\sigma /{\sigma }_{\max }$$

In Equation (4), N denotes the number of points analyzed along the assessment line AB at each cross section.

### Estimation of Switching Length and Time using the SI

**Figure 3:**
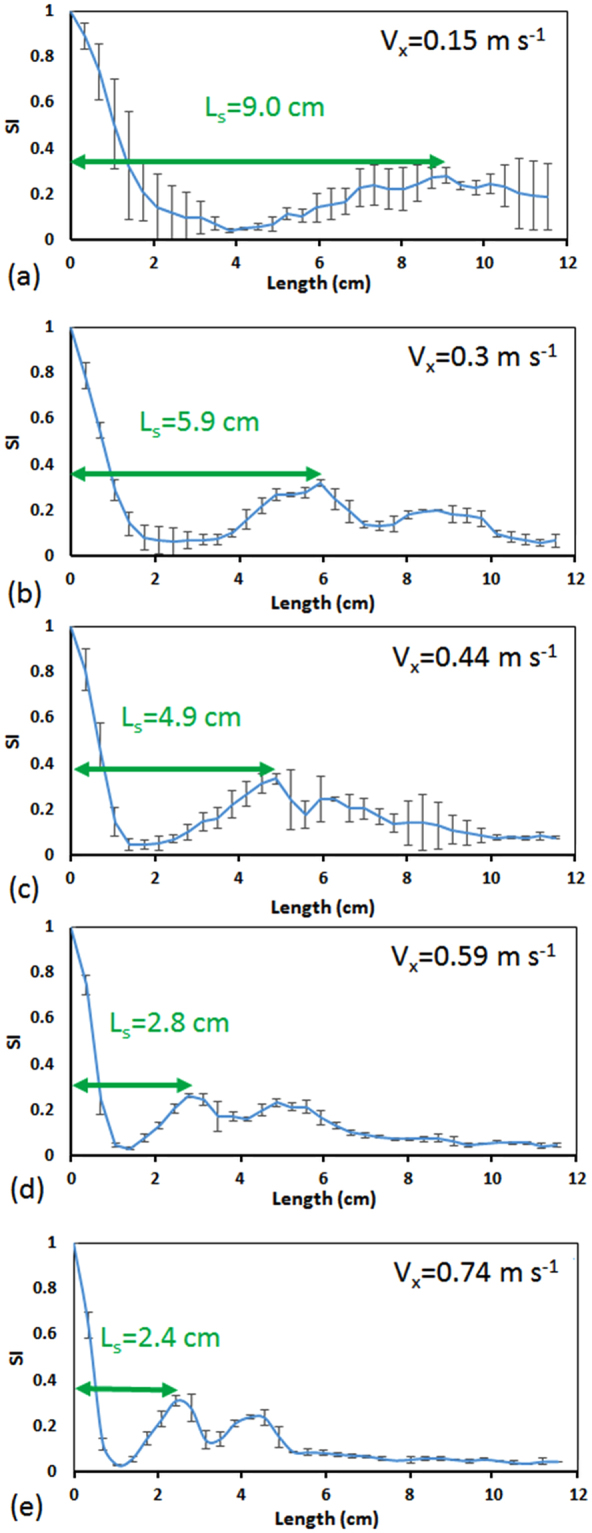
Switching Index (SI) diagrams along the length of the channel at various inlet axial velocities, V_x_, of methylene blue and water that were co-injected into a device with R = 2 cm and cross section area of 150 µm × 150 µm. SI decreases along the channel as the two solutions form counter-rotating vortices and increases when they start separating from each other into distinct phases again downstream the channel. The first peak in the SI plot indicates the exact location of the first switch in position (L_s_) of methylene blue and water solutions (i.e., 180° recirculation). At higher axial velocities, a second switch corresponding to a full 360° recirculation of fluids can be clearly seen. Error bars represent the standard deviation of three experimental repeats.

We first investigated the suitability of the SI in Equation (5) for investigating the Dean flow-based recirculation of fluids and quantifying the switching length (L_s_) and time (t) at which water and methylene blue solutions exchange radial positions in a curved microchannel. For this, we conducted a series of experiments with the microfluidic device that had a radius of curvature of R = 2 cm and cross-sectional dimensions of 150 µm × 150 µm. Methylene blue and water solutions were co-injected into the device at various axial velocities (V_x_ = 0.15–0.74 ms^−1^) and the effect of fluid axial velocity on SI was investigated along the channel length. The results are shown in Fig. 3.

**Figure 4:**
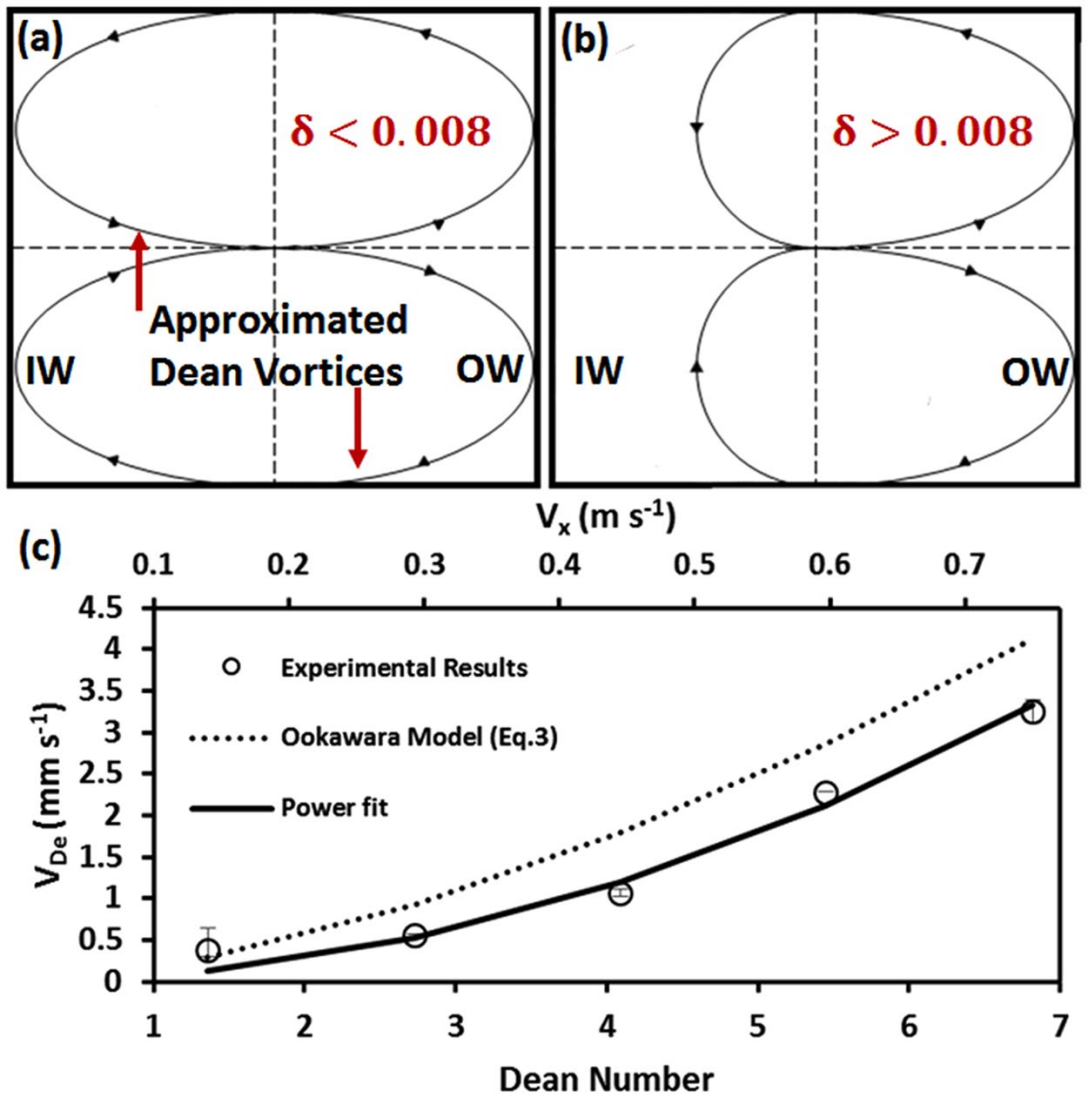
Dean vortex shape approximation and calculated average Dean velocities in a curved microchannel with R = 2 cm and cross section area of 150 µm × 150 µm. Dean vortices were assumed to follow (**a**) an elliptical path for *δ* < 0.008 ($\delta =D/2R$) and (**b**) a half-elliptical half-circular path for *δ* > 0.008^10^. (**c**) The switching lengths obtained experimentally and the approximated average lateral travel distances were used to calculate the average Dean velocities (V_De_) at different axial velocities in the abovementioned device. The solid line in (**c**) shows a power function (V_De_ = aDe^b^) fitted over the experimental data points with constants a = 0.072 and b = 2 (R^2^ = 0.98). The dashed line in (**c**) shows the Ookawara’s^30^ equation for Dean velocity (mm s^−1^) with a = 0.18 and b = 1.63 (R^2^ = 0.71).

As shown in Fig. 3, the *SI* was equal to unity at the entrance of the channel ($\sigma ={\sigma }_{max}$ in Equation (5)) where the methylene blue and water solutions were completely separated (Fig. 2a-1) and the intensity curve was similar to a step function (Fig. 2b-1). Moving along the length of the channel at each velocity setting, the *SI* decreased gradually as a result of Dean flow-based recirculation of fluids in the channel (Fig. 2a-2 and b-2). The minimum SI value in each plot was obtained when the water stream was completely sandwiched in between two methylene blue streams at its top and bottom sides (Fig. 2a-3). This condition resulted in a flat intensity diagram (Fig. 2b-3) and minimization of $\sigma$ in Equation (4). Afterwards, the *SI* started increasing to its first peak value due to continuation of fluids recirculation that resulted in separation of methylene blue and water solutions into two phases in the radial direction of the channel again (180° recirculation in Fig. 2a-4 and b-4). The peak *SI* value was not equal to the initial unity because of two reasons; first, minor diffusion taking place along the interface of water and methylene blue solutions and second, various fluid particles having different local Dean velocities at different locations along the cross section of the channel. These factors prevented achieving a complete switch in fluids’ position with an interface as distinct as the one at the channel entrance. They led to an overall reduction of *c*_*i*_ and $\bar{c}$ values in Equation (4) at the location of the switch, hence a smaller *SI* value in Equation (5). Although these factors resulted in reduction of *SI* at the first peak, but the switching location (L_s_) could still be determined from the *SI* plot which was later used to calculate the average Dean velocity in the channel. By moving further downstream in the channel, fluid recirculation continued to occur and dependent on the fluid velocity, the *SI* curves either demonstrated a second peak corresponding to a second switch of position or a plateau that indicated indistinguishable mixing of fluid layers in the channel^7,18^.

As mentioned above, the first peak on the $SI\,$diagrams corresponded to the exact switching location of methylene blue and water solutions in the channel which is denoted by the switching length, L_s_, in Fig. 3. With increasing the axial velocity of the fluid from 0.15 m s^−1^ to 0.74 m s^−1^ (De = 2.73–6.82), we clearly observed that the fluid recirculation became stronger and the $SI$ peaks started to shift towards the left of the diagrams corresponding to reduction of switching length from L_s_ = 9 cm (at V_x_ = 0.15 m s^−1^) to L_s_ = 2.4 cm (at V_x_ = 0.74 m s^−1^). We used the switching length L_s_ and the corresponding axial velocity V_x_ to calculate the time of the first switch (t = L_s_/V_x_). We also identified appearance of a second switch (i.e., complete 360° recirculation at the 2^nd^ peak on SI diagrams in Fig. 3e) as the axial velocity was increased in the channel to 0.59 m s^−1^ and beyond. This was because the vortices got stronger at higher De numbers and switching length decreased resulting in an opportunity for the fluids to show a complete recirculation in the device. Further switches were not identifiable with our quantitative *SI*-based approach either due to full mixing of the fluids or enhanced 3D complexity in layered structure of the fluids that could no longer be distinguished with our top view-based imaging method.

### Estimation of Average Lateral Travel Distance and Dean Velocity

To approximate the average Dean velocity (V_De_) from the experiments discussed in the previous section, an estimation of the average lateral travel distance by the fluid particles (L_R_) was needed. In this case, V_De_ can be calculated from Equation (6) using the experimentally determined switching time in the previous section.

6 $${V}_{De}=\frac{{L}_{R}}{t}=\frac{{L}_{R}{V}_{x}}{{L}_{s}}$$

The average lateral travel distance was approximated at 0.75D by Ookawara *et al*.^30^ by assuming an simplified rectangular streamline shape for Dean vortices. However, Martel and Toner^10^ showed that the channel curvature ratio $(\delta =\frac{D}{2R})$ is a key parameter for determination of the shape of Dean vortices. Their simulations (confirmed by our numerical results) indicated that for curvature ratios smaller than 0.008, fluid particles tend to follow elliptical streamlines such as the one shown in Fig. 4a. However, for $\delta$ values greater than 0.008, the bulk of the laterally-flowing fluid shifts towards the outer wall of the channel, leading to formation of asymmetric oval shape streamlines for Dean vortices. Hence, the fluid elements closer to the inner wall of the channel participate less in the circulatory motion. Accordingly, we approximated our Dean vortices with elliptical shape streamlines at low $\delta$ (<0.008 in Fig. 4a), while a half-circle half-ellipse shape (Fig. 4b) was used to approximate the lateral travel distance, L_R_, of fluids in channels with higher curvature ratios ($\delta$ > 0.008)$.$ Average Dean velocities for the experiments presented in Fig. 3 were then calculated based on Equation (6) using the approximated L_R_ values discussed above and the results are presented in Fig. 4c.

As demonstrated in Fig. 4c, for a curved microchannel with R = 2 cm and cross section of 150 µm × 150 µm (elliptical streamline model with $\delta =0.0038$), by increasing the axial velocity of the fluid from 0.15 m s^−1^ to 0.74 m s^−1^, the average Dean velocity increased from V_De_ = 0.57 mm s^−1^ to V_De_ = 3.25 mm s^−1^, respectively. This clearly supports the discussion of faster switching of fluids at higher De numbers in a curved microchannel. Following the proposed power equation type (*V*_*De*_ = *aDe* ^*b*^) by Ookawara *et al*.^30^, we fitted a power function to our calculated V_De_ values in Fig. 4c. The experimentally-driven constants of the fitted equation were *a* = *0.07*2 and *b* = 2 (*R* ^2^ = *0.98*) which were different from the numerical values reported by Ookwara *et al*.^30^ (i.e. *a* = *0.18* and *b* = *1.63*, *R* ^2^ = *0.7*1).

The power dependence of V_De_ on the Dean number has been confirmed by others^10^ while the disagreement between Ookawara’s equation and case-specific models has been reported by researchers such as Guan *et al*.^45^ These differences may be originated from differences in fluid properties and or dimensional variations among different microchannels. Having shown that our methodology has the capability of characterizing the average Dean velocity in a curved microchannel, we continued our investigations by examining the effects of other important parameters such as the radius of curvature, hydraulic diameter, width, height, and viscosity on Dean velocity using a combination of complementary experimental and numerical approaches. The objective of these parametric studies was to explore if an inclusive equation could be proposed for estimation of average Dean velocity in curved microfluidic channels.

### Effect of Channel Radius of Curvature on Dean Velocity

**Figure 5:**
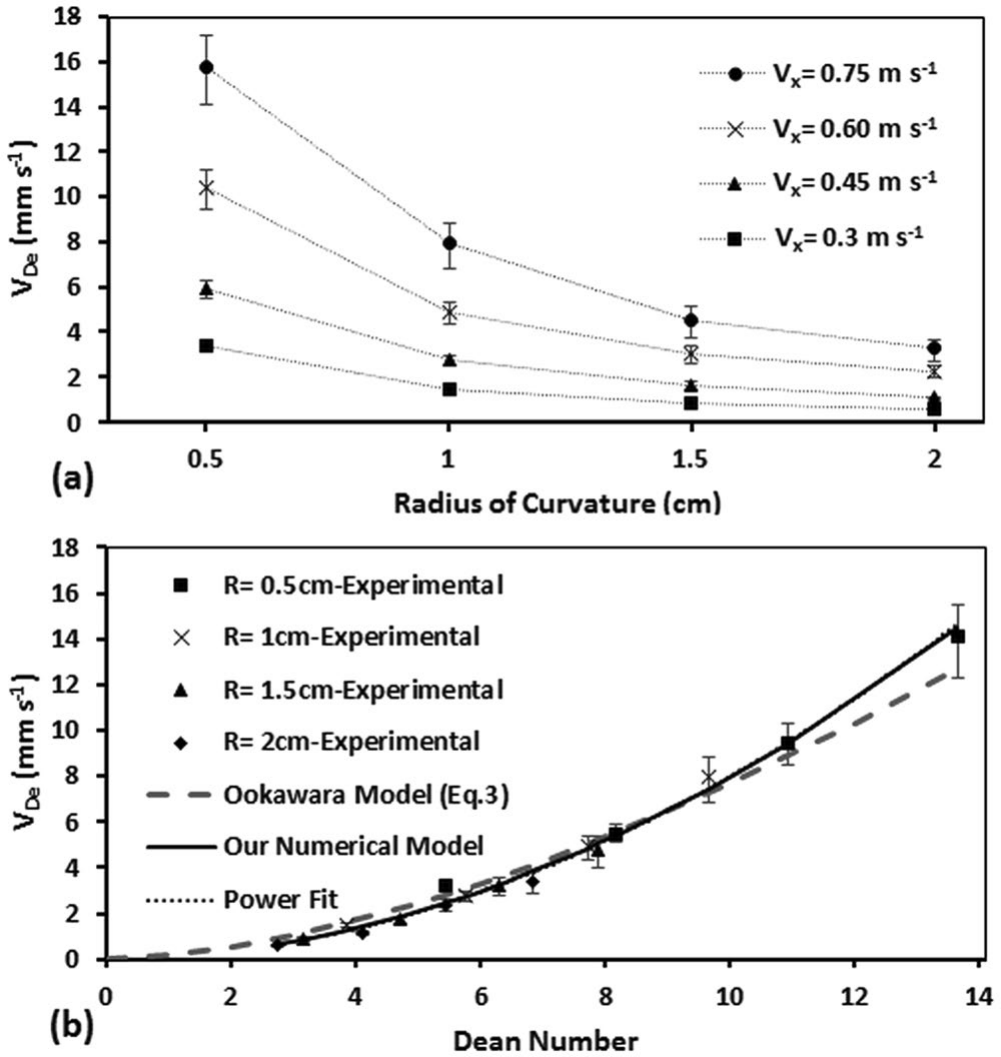
Effect of radius of curvature of the channel (R = 0.5–2 cm) on Dean velocity shown (**a**) at various axial velocities and (**b**) using the non-dimensional Dean number for devices with 150 µm × 150 µm cross section. The Dean velocity decreases as the radius of curvature increases at each inlet velocity. Increasing the Dean number (via axial velocity) for each device resulted in an increase in Dean velocity. A power function (V_De_ = aDe^b^) fitted over the experimental results in (**b**) provided the constants of a = 0.090 and b = 1.95 with R^2^ = 0.98. Numerical results based on our model (a = 0.096 and b = 1.92) and Ookawara’s model (a = 0.18 and b = 1.63) are also shown with a solid and a dashed line, respectively.

Here, we examined the effect of the radius of curvature of the microchannel on Dean velocity. For this purpose, curved microchannels with the same cross section area of 150 µm × 150 µm but various radii of curvature of R = 0.5, 1, 1.5, and 2cm were fabricated and tested at various axial flow velocities (V_x_ = 0.15–0.74 m s^−1^). The results of this study are shown in Fig. 5a. As demonstrated, at a constant axial velocity, an increase in the radius of curvature resulted in reduction of Dean velocity due to lowering of the Dean vortex strength. The effect of radius of curvature was more strongly observed at higher axial velocities. We also plotted the obtained V_De_ values in the above set of experiments as a function of Dean number in Fig. 5b.

For devices that had different radii of curvatures but approximately the same Dean number, we observed a highly similar Dean velocity as shown in Fig. 5b. A power function (*V*_*De*_ = *aDe* ^*b*^) was fitted to the overall results in Fig. 5b (dotted line) and the correlating constants of *a* = *0.090* and *b* = *1.95* (*R* ^*2*^ = *0.98*) were obtained. The Dean velocities from devices with various radius of curvature followed this power correlation precisely, indicating that the effect of radius of curvature is sufficiently captured by the R-parameter already included in the De number. For comparison purposes, the Ookawara’s equation is also plotted in Fig. 5b (dashed line). With a R^2^ value of 0.94, it overpredicted the experimental Dean velocities at De < 8 and underestimated them at higher De values. We hypothesize that the mismatch between Ookwara’s equation and our experimental results in Figs 4c and 5b might stem from the differences between the geometrical dimensions of the curved microchannels and approximated lateral travel distances used in both studies. Therefore, we decided to develop and validate a numerical model to further investigate the effect of other parameters on Dean velocity.

### Numerical Model to Investigate Dean Velocity Parametrically

As discussed in the Methods section and Supplementary File 1, we used COMSOL Multiphysics to simulate the Dean flow in our microchannels and obtain the Dean velocities accordingly. The experimental conditions reported in Fig. 5b were simulated and the numerical results are plotted with a solid black line in Fig. 5b for comparison purposes. The results followed a power function trend (R^2^ = 0.98) with *a* and *b* coefficients of 0.096 and 1.92, respectively, that were slightly different from the power function fit to our experimental results. This verified that our model could predict Dean velocities with a better precision when compared to the model reported by Ookawara *et al*.^30^. This better precision stems from higher similarity between our numerical simulation and experiments in terms of geometry and flow conditions as well as a better approximation for lateral travel distance of fluid particles as shown in Fig. 4a and b.

The power functions reported in this paper and by other researchers for estimation of Dean velocity are limited solely to parameters involved in the Dean number with fixed powers. We hypothesized that factors such as specific channel geometries (i.e. width vs. height) and fluid viscosity may have significant effects on Dean velocity that available power functions cannot predict accurately. To investigate this, we used our validated numerical model to examine the effect of these parameters on Dean velocity.

### Effect of Hydraulic Diameter on Dean Velocity

**Figure 6:**
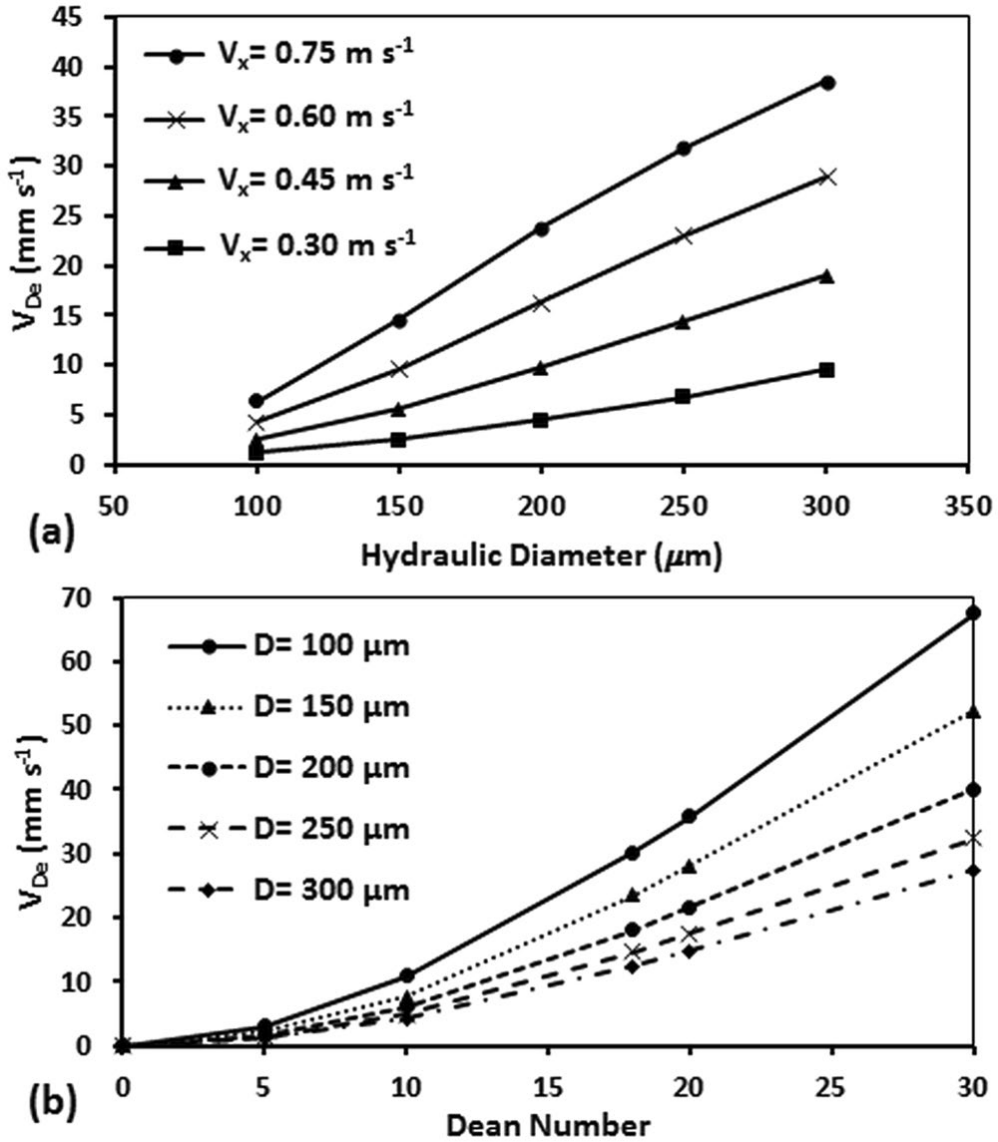
Effect of channel hydraulic diameter (D) on average Dean Velocity (V_De_) using numerical modeling of a curved microchannel with R = 0.5 cm radius of curvature shown (**a**) at various axial velocities and (**b**) using the non-dimensional Dean number. At each inlet velocity, increasing the hydraulic diameter resulted in higher V_De_. But when plotted V_De_ as a function of De in (**b**), we observed that the hydraulic diameter inversely affects the V_De_ when Dean number is kept constant. Single a and b constants could not be found to predict V_De_ with a single power function (V_De_ = aDe^b^).

The effect of hydraulic diameter of the channel (D) on Dean velocity is captured by a power of 2.88 in our preliminary model presented in previous section (*V*_*De*_ = *aDe* ^*b*^, *a* = *0.096* and *b* = *1.92*). Here, we were interested to investigate whether this power sufficiently captures the effect of hydraulic diameter on Dean velocity. For this purpose, we performed simulations on square cross-section microchannels with R = 0.5 cm and hydraulic diameters of 100 µm-300 µm at 0 < De < 30. We already showed in Fig. 5b that at a constant De number, the radius of curvature does not affect Dean velocity significantly. Hence, the smallest radius of curvature was selected for numerical investigations of hydraulic diameter which reduced the number of mesh elements and time of computation considerably. Results of these simulations are presented in Fig. 6a. As demonstrated, at a constant axial velocity, an increase in the hydraulic diameter of the channel resulted in an increase in the Dean velocity due to stronger secondary vortices (i.e. higher De numbers). The resulted Dean velocities in the numerical simulations above were also plotted as a function of Dean number in Fig. 6b.

As shown in Fig. 6b, when De number increases at a constant hydraulic diameter, the Dean velocity also increases due to formation of stronger secondary vortices. The results followed a power function (*V*_*De*_ = *aDe* ^*b*^) similar to what we observed in Figs 4c and 5b. However, a single power function with the same coefficients *a* and *b* could not be fitted over all the data points, suggesting that the hydraulic diameter parameter with a fixed power could not thoroughly capture the effect of channel geometry on Dean velocity. In other words, increasing the hydraulic diameter while keeping the De number constant resulted in reduction of the Dean velocity which completely contradicts with predictions provided by the power functions presented above or used in various forms in the literature.

The hydraulic diameter is derived from the width and height of the channel. Hence, to further investigate the effect of channel geometry on Dean velocity, we continued our studies with modeling of a series of channels with different widths and heights in the following section.

### Effect of Channel Width and Height on Dean Velocity

**Figure 7:**
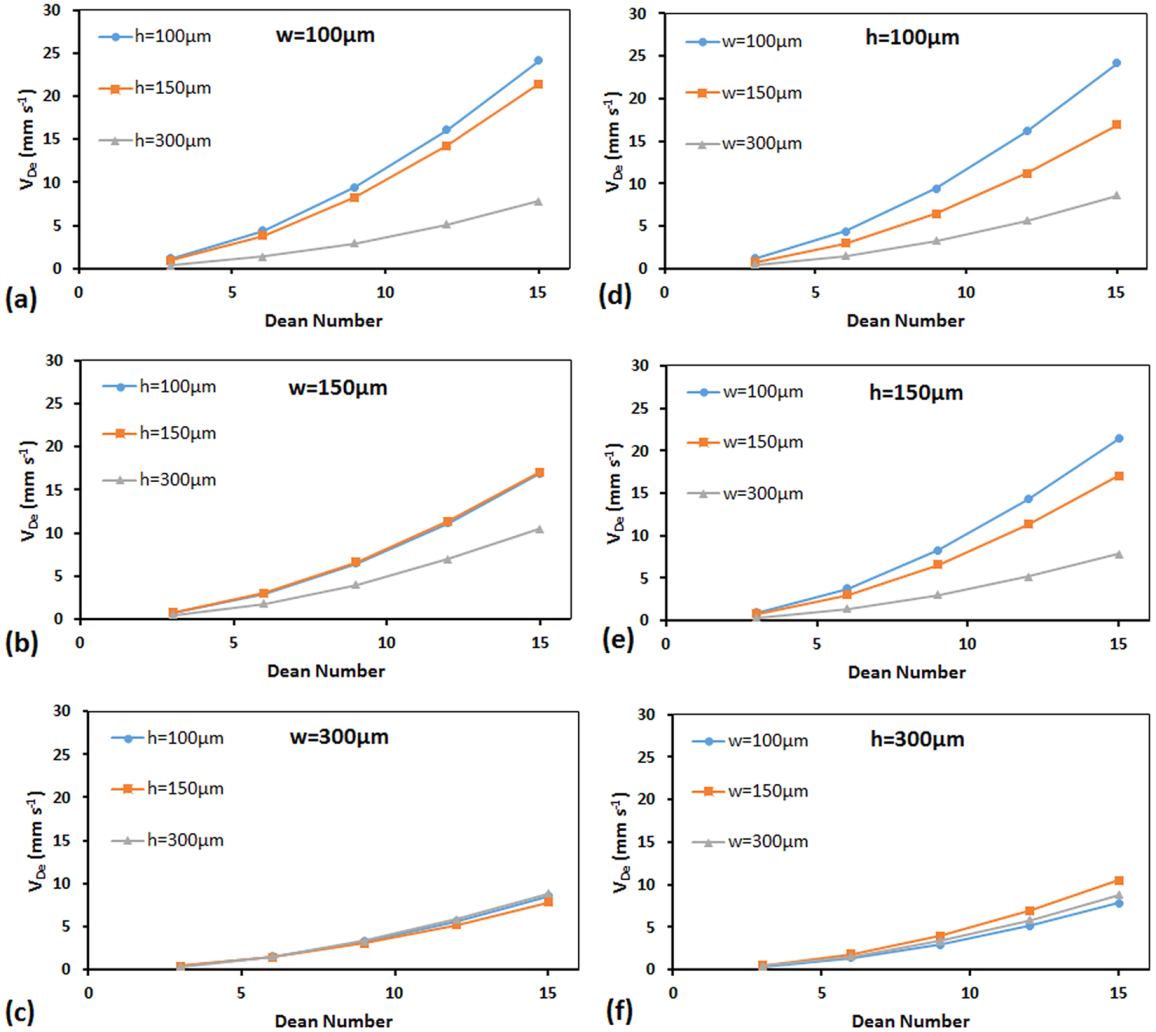
Effect of channel width (w) and height (h) on Dean velocity for curved microchannels with R = 0.5 cm. (**a**–**c**) show the effect of height at fixed channel widths while (**d**–**f**) show the effect of width at fixed channel heights on Dean velocity.

The effect of channel geometry was further investigated by examining the roles of width (w) and height (h) of the channel individually on Dean velocity. Our strategy was to simulate 0.5 cm radius of curvature curved microchannels with square and rectangular cross sections that had widths and heights of 100 µm, 150 µm and 300 µm. The resultant Dean velocities for different combinations of channel widths and heights at various De numbers are plotted in Fig. 7.

As shown in all plots of Fig. 7, increasing the De number at a constant channel width and height expectedly resulted in an increase of Dean velocity. At a constant De number, both height and width had an inverse effect on Dean velocity as shown in Fig. 7(a–c) and (d–f), respectively. For instance, increase of channel height from 100 µm to 150 µm to 300 µm at a constant w = 100 µm (Fig. 7a) and De = 15 resulted in a decrease of Dean velocity from V_De_ = 24.2 mm s^−1^ to V_De_ = 21.5 mm s^−1^ to V_De_ = 7.8 mm s^−1^, respectively. This is because at a constant w, the higher h corresponds to a longer distance that fluid elements should travel in a vortical motion, which results in a lower V_De_. However, as the width of the channel became larger, the effect of height on Dean velocity became less dominant. For example, in the 300 µm-wide microchannel (Fig. 7c), changes in height did not significantly affect Dean velocity at any constant De number. Similar trends described above for height were also seen for the effect of width of the channel on Dean velocity as demonstrated in Fig. 7(d–f). Increase of width at a constant height decreased the Dean velocity when De number was maintained constant. However, this decreasing trend became less significant as the height of the channel increased from h = 100 µm (Fig. 7d) to h = 300 µm (Fig. 7f).

The overall behavior can be explained by introducing the larger dimension of the channel as the determinative parameter influencing the Dean velocity. Channels with equal larger dimension possessed Dean velocities very close to each other and variation in the smaller dimension of the channel had low to no effect on the Dean velocity in all cases. This makes a logical sense because the larger dimension of the channel determines the distance along which fluid particles must travel mostly with their assumed Dean velocities. The results of this section suggested that an additional factor representing the inverse effect of the largest dimension of the microchannel on the Dean velocity must be included in the comprehensive correlation that we aim to derive for V_De_.

### Effect of Kinematic Viscosity on Dean Velocity

**Figure 8:**
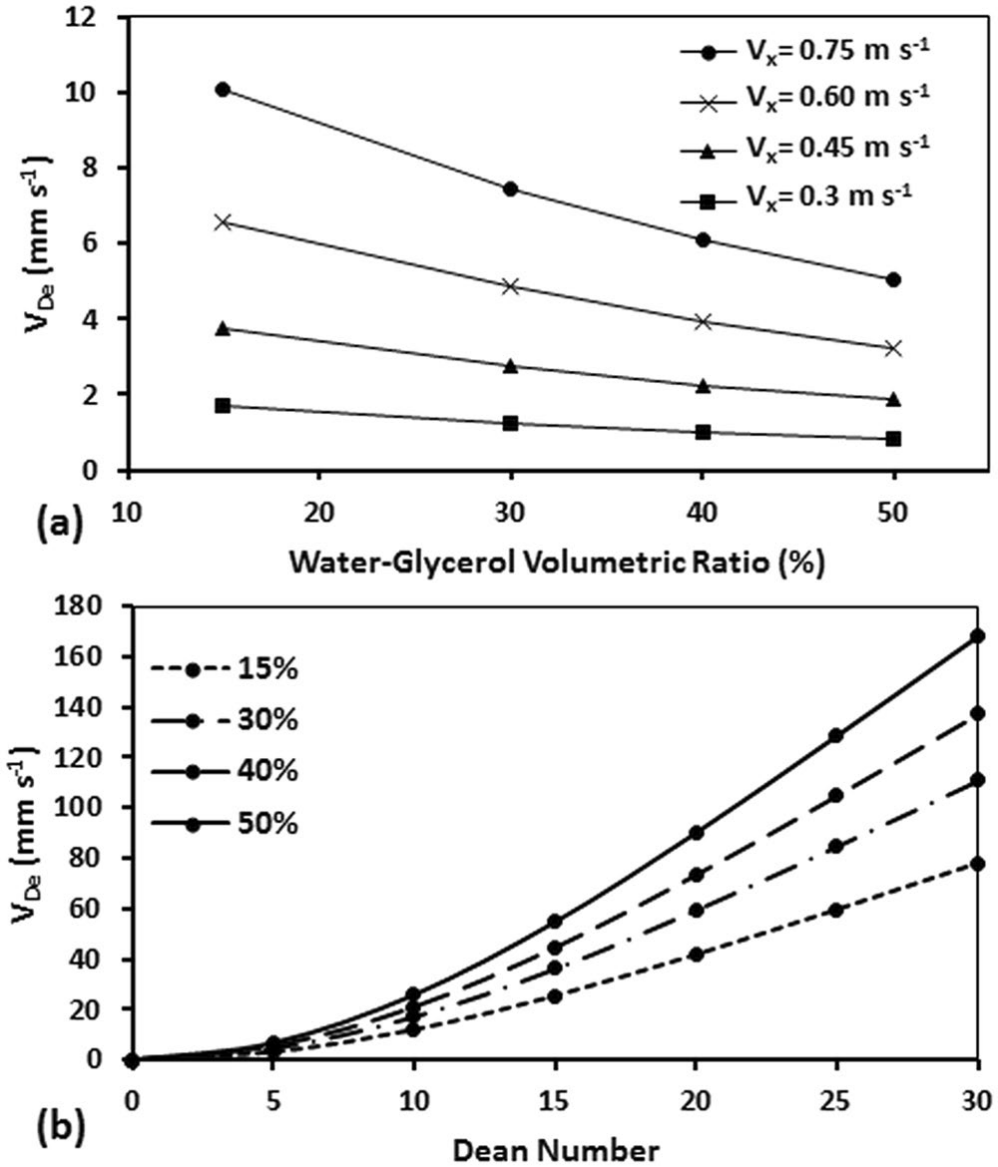
Effect of fluid kinematic viscosity on Dean velocity in a 150 µm × 150 µm curved microchannel with R = 0.5 cm shown (**a**) at various axial velocities and (**b**) using the non-dimensional Dean number. Increasing the viscosity while axial velocity is constant caused a reduction in V_De_. However, when Dean number was kept constant, viscosity directly affected the V_De_. Single a and b constants could not be found to predict V_De_ with a single power function (V_De_ = aDe^b^).

Having studied the effects of various channel geometries on Dean velocity, we became interested in investigating whether the kinematic viscosity parameter in the De number is sufficient to capture the effect of this variable on Dean velocity. In other words, we wanted to understand if changing the viscosity while keeping the De number constant results in any change in the Dean velocity. Therefore, using our numerical model we simulated a microfluidic device with R = 0.5 cm radius of curvature and cross-sectional dimension of 150 µm × 150 µm. We increased the kinematic viscosity of the fluids from $\nu ={10}^{-6}$ m^2^ s^−1^ to $\nu =3.21\times {10}^{-6}$ m^2^ s^−1^ corresponding to 0–50% volumetric water-glycerol mixtures based on the results of Cheng^46^. Results of these simulations are presented in Fig. 8a. As demonstrated, at a constant axial velocity, an increase in the viscosity of the fluid resulted in reduction of the Dean velocity due to weakening of the secondary vortices (i.e. lower De numbers). Dean velocities as a function of De number are also plotted in Fig. 8b for the above experiments.

As shown in Fig. 8b, an increase in the De number at each viscosity level causes the Dean velocity to increase by following a power function (*V*_*De*_ = *aDe* ^*b*^) as observed for other parameters above. However, two fixed values for *a* and *b* could not be found to represent all data points with a single power function. Thus, we concluded that the effect of kinematic viscosity should be included further in the final correlation that we aim to derive for V_De_.

### Non-Dimensional Dean Velocity Correlation

**Figure 9:**
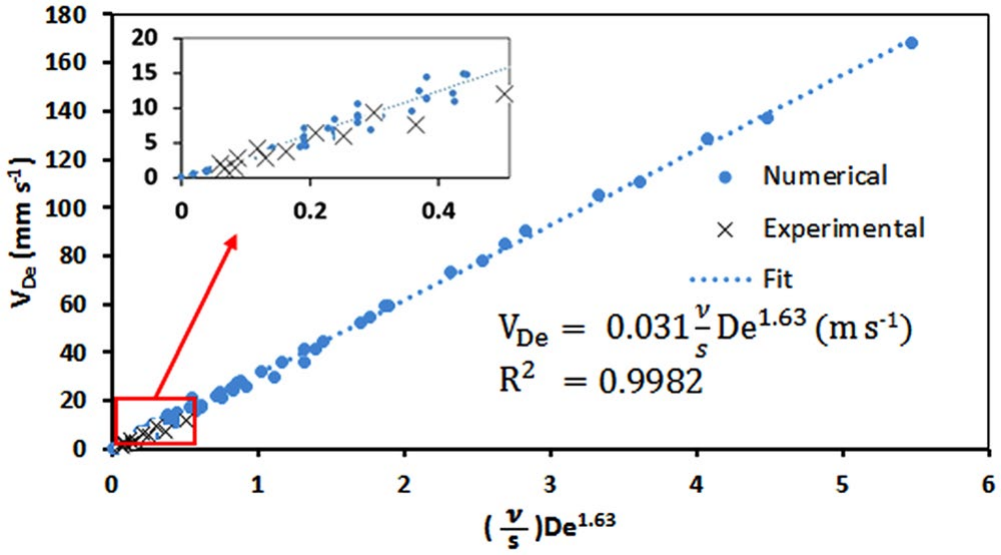
Dean velocity plotted against $(\frac{{\boldsymbol{\nu }}}{{\boldsymbol{s}}}){D}{{e}}^{1.63}$ based on all numerical results obtained in this study. A linear function could be fitted over the data points (R^2^ = 0.9983) with a = 0.031 as the constant of linearity. The inset figure shows our experimental Dean velocities from single experiments in two devices with cross sectional dimensions of 100 µm × 150 µm and 300 µm × 150 µm (cross data points) that follow the numerically-determined fit very well.

Our experimental and numerical results in the previous sections along with other researchers’ work has led us to conclude that Dean velocity has a power-function dependency on the De number. However, the existing functions presented in the form of *V*_*De*_ = *aDe* ^*b*^ are not able to represent the effect of some parameters such as the largest channel dimension (s) at all, while not sufficiently capable of capturing the effect of some other parameters such as kinematic viscosity (υ). Here, we propose that these parameters should be added to the above formula (i.e. Equation (7)) in order to obtain a more comprehensive correlation for estimation of Dean velocity in curved microchannels.

7 $${V}_{De}=\,a{(\frac{\nu }{s})}^{n}D{e}^{b}$$

where *a*, *b* and *n* are the correlation constants that were needed to be determined. In order to find these unknowns, Dean velocities as a function of ${(\frac{\nu }{s})}^{n}D{e}^{b}$ were plotted using all of our numerical results presented before (see Supplementary Fig. S2). The *n* and *b* values were varied in the range of 0.25–2.5 and the best fits to our data points were investigated. For keeping a similarity with the commonly-known Dean power in the literature, we intentionally set *b* = *1.63*. The studies then showed that by setting *n* = *1*, the numerical Dean velocities will become linearly dependent on $(\frac{\nu }{{\rm{s}}}){{\rm{De}}}^{1.63}$ with a constant of linearity of *a* = *0.031* as shown in Fig. 9 (best fit with R^2^ = 0.9983).

Based on the studies above, the final correlation for the average Dean velocity in a curved microchannel can be presented as:

8 $${V}_{De}=\,0.031(\frac{\nu }{s})D{e}^{1.63}$$

Equation (8) can be rearranged to provide a fully dimensionless relationship between Reynolds number based on average Dean velocity and largest dimension of the channel (called lateral Re number) and the De number based on axial velocity.

9 $$R{e}_{s,{V}_{De}}=0.031\,D{e}^{1.63}$$

Lastly, the suitability of the proposed correlation was experimentally validated. For this purpose, we conducted a new set of experiments in devices with R = 1 cm and cross-sectional dimensions of 100 µm × 150 µm and 300 µm × 150 µm (see Supplementary Figs S3,S4). We also conducted experiments with a 15% volumetric water-glycerol mixture in a device with R = 0.5 cm and cross-sectional dimension of 150 µm × 150 µm (see Supplementary Fig. S5). The resultant experimental Dean velocities are plotted in the inset of Fig. 9. As shown, the proposed correlation in Equation (9) was accurate enough for estimation of Dean velocities in these experiments.

## Conclusion

In this study, we developed experimental and numerical models to examine the effects of radius of curvature, hydraulic diameter, and width and height of the channel as well as the kinematic viscosity of the fluid on Dean velocity in curved microchannels. We showed that Dean velocity cannot be merely estimated with a widely-used power function (*V*_*De*_ = *aDe* ^*b*^) because this correlation fails to predict the effects of the larger dimension of the channel and kinematic viscosity on Dean velocity. Instead, we proposed a semi-empirical correlation that relates the lateral Reynolds number of the channel (based on V_De_ and the largest channel dimension) to the Dean number. This correlation was accurate enough to predict the average Dean velocity for Dean numbers lower than 30 for various experimental conditions. We envision the widespread use of this correlation in estimation of Dean velocities in curved and spiral microchannels used widely in sample processing and preparation applications.

## Methods

### Device Fabrication

Standard photolithography^47^ and soft lithography^48^ methods were used to fabricate the abovementioned microfluidic devices. In order to prepare the master replication molds, negative SU8 2075 photoresist (Microchem Corp., MA, USA) was spun over 4-in diameter silicon wafers which were obtained from University Wafers Corp. (MA, USA). Coated wafers were then prebaked at 65 °C for 5 minutes and 95 °C for 30 minutes followed by exposure to ultraviolet light through a photomask. Post-bake treatment was carried out at 65 °C and 95 °C for 5 and 12 minutes, respectively. The process was finalized by dissolving the unexposed SU8 using SU8 developer solution. Subsequently, degassed mixture of 10:1 ratio PDMS pre-polymer base and curing agent (Sylgard 184 kit, Dow Corning, MI, USA) was poured over the mold and heated at 80° for 2 hr. The cured PDMS layer was peeled off the master mold and holes were punched on it at inlets and outlets of the microchannel. In order to enclose the microchannel, an oxygen plasma machine (Harrick Plasma, PDC-001, NY, USA) was used to bond cured PDMS layers with glass slides at 45W for 30–35s, followed by heating at 80° for 5–10 minutes to enhance the bonding. To prepare the device for experiments, Tygon tubes (Saint-Gobain, Paris, France) were connected to the punched inlets and outlets of the bonded device.

### Experimental Setup and Procedures

Two 10 mL syringes containing Methylene Blue (MB) dyed and tap water were installed onto a dual syringe pump (Legato 110, KD Scientific, USA) and used to co-flow the fluids with favorable flow rates into the microfluidic device that was positioned under a microscope for optical imaging. In order to investigate the effect of viscosity on Dean velocity, we used water and glycerol mixture with 15% volumetric ratio while viscosity and density values of the mixture were calculated from the work of Cheng^46^. Inlet flow rate was changed from 0.2–1 mL min^−1^ (corresponding to axial velocity of V_x_ = 0.15–0.74 m s^−1^ and Re = 22.5–112.5) and flow was allowed to stabilize in the devices for 2 min. After flow stabilization, a video from the entire channel, from inlet to outlet, was recorded at a 5x magnification via a camera (Point Grey, BC, Canada) connected to the inverted microscope (Bioimager, ON, Canada). Devices were properly washed after each experiment with water for at least 5 minutes to remove any MB residue. Experiments were repeated at least three times for each flow rate and viscosity setting to obtain average and standard deviation values at each experimental condition.

### Numerical Model

We used COMSOL Multiphysics to simulate the above-mentioned curved microchannels used in our experiments. For further information about the model, please see Supplementary File 1. Approximately 10^5^–10^6^ mesh elements were used in each simulation depending on the size of the microchannel to make our simulations mesh-independent. Inlet velocity and atmospheric pressure boundary conditions were employed for the inlets and outlets, respectively. The channel walls were all set to no-slip boundary condition. The average V_De_ was calculated by deriving the average tangential velocities over the cross section of the channel normal to the wall. We decreased the size of the modeled channel to a shorter length to enhance our computation speed and accuracy. The average V_De_ along the channel was calculated and no significant changes in V_De_ was observed after 60°, so we modeled only a 60° portion of the channel. In order to prevent the outlet boundary condition from affecting the results, we calculated the average V_De_ at a cross-section that was located 10° before the outlet.

### Data availability

The datasets generated and/or analyzed during the current study are available from the corresponding author on reasonable request.

## Electronic supplementary material

Supplementary File

Video S1

Video S2
