# Supplementary material for: Semi-Empirical Estimation of Dean Flow Velocity in Curved Microchannels
Source: Sci Rep. 2017 Oct 20;7:13655. doi: 10.1038/s41598-017-13090-z (PMC5651805; doi:10.1038/s41598-017-13090-z)
Supplement: Supplementary file 1 — Supplementary File [file 41598_2017_13090_MOESM1_ESM.doc]

Supplementary Materials

**Semi-Empirical Estimation of Dean Flow Velocity in Curved Microchannels**

Pouriya Bayat and Pouya Rezai*

Department of Mechanical Engineering, York University, Toronto, ON, Canada

* Corresponding Author: Pouya Rezai, BRG 433B, 4700 Keele St, Toronto, ON, M3J 1P3, Canada; Tel: 416-736-2100 ext. 44703; Email: [prezai@yorku.ca](mailto:prezai@yorku.ca)

**1. Details of Numerical Model**

All of the simulations were performed using the laminar flow module of COMSOL Multiphysics. The 3D geometry of the model consisted of a 60 degree portion of a curved microchannel with a specific width and height (Fig. S1a). Dynamic viscosity and density of water were set to 0.001 Pa.s and 1000 kg.m^-3^, respectively. The corresponding properties of water-glycerol mixtures were extracted from the work of Cheng^1^ and set manually in our model. The inlet, outlet and wall boundary conditions were set as flow rate (0.2-1 ml/min), atmospheric pressure and no slip boundary conditions, respectively. We used COMSOL Multiphysics’ mesh module to mesh the geometry with triangular elements (Fig. S1b). The number of mesh elements varied from 10^5^ to 10^6^ depending on the hydraulic diameter of the channel. Also, the number of the elements increased closer to the channel wall to be able to compensate for the severe gradients in the boundary layer region. We used a linear solver with iterative method (GMRES) left preconditioning with maximum iteration number of 200 to solve the Navier-Stokes equation for velocity and pressure as dependent variables (Fig. S1c and S1d). Upon solving the equations, a cross section of the channel located at 10 degrees before the outlet was analyzed and the average lateral velocities over this cross section were calculated and reported as the average Dean velocities (V_De_).


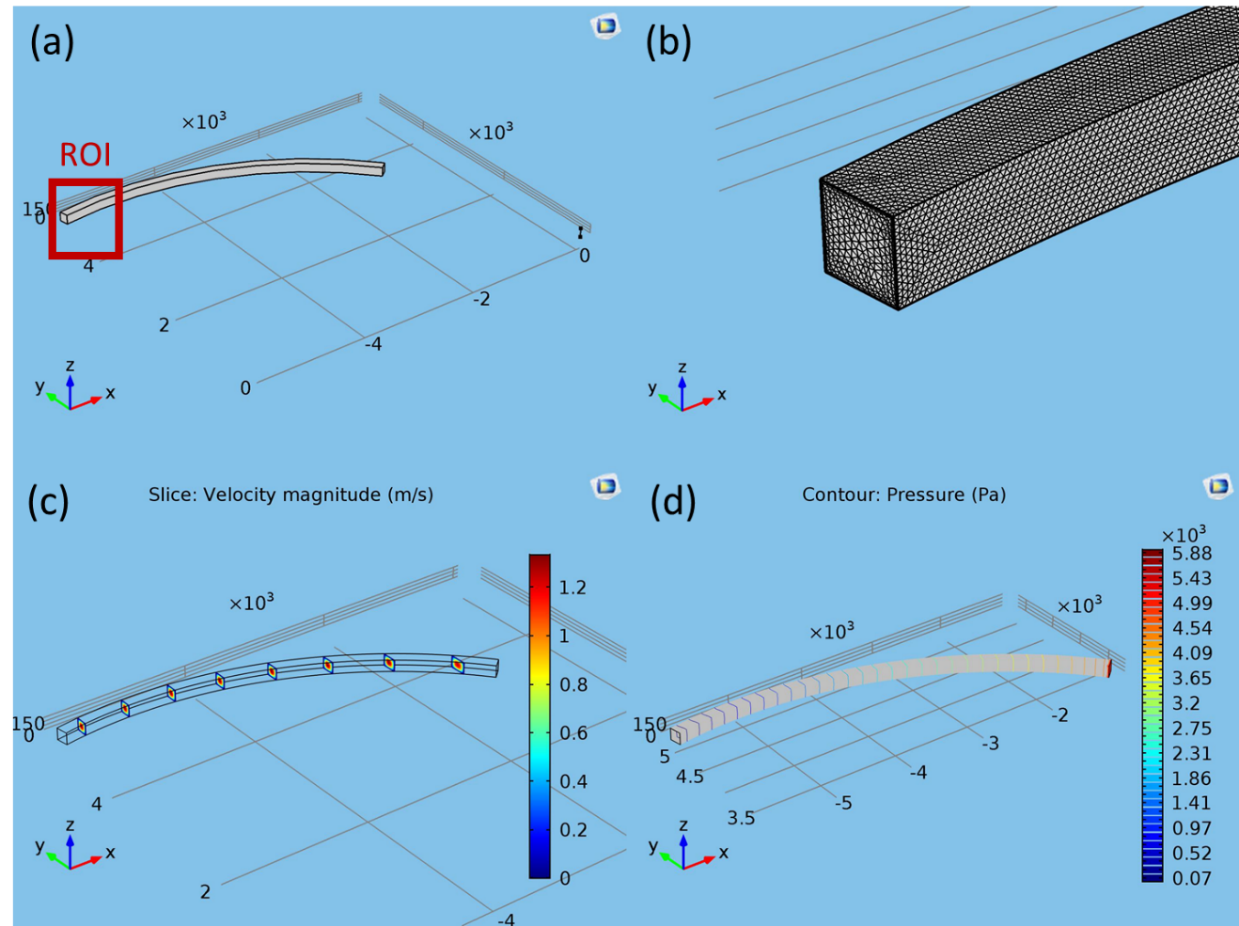


*Fig. S1. Numerical model used for determination of Dean velocity. (a) Geometry of a microchannel with R=0.5 cm and cross-sectional dimension of 150µm×150µm. (b) Mesh elements in the Region Of Interest (ROI) shown in (a). The number of mesh elements increased at the wall. Total number of elements were approximately 8×10^5^. (c) Velocity distribution over yz-planes for water at De=12. (d) Pressure contours for the geometry presented in (a) and the flow conditions mentioned in (c)*.

**2. Detailed Comparison of Dean Velocity between Numerical and Experimental Results**


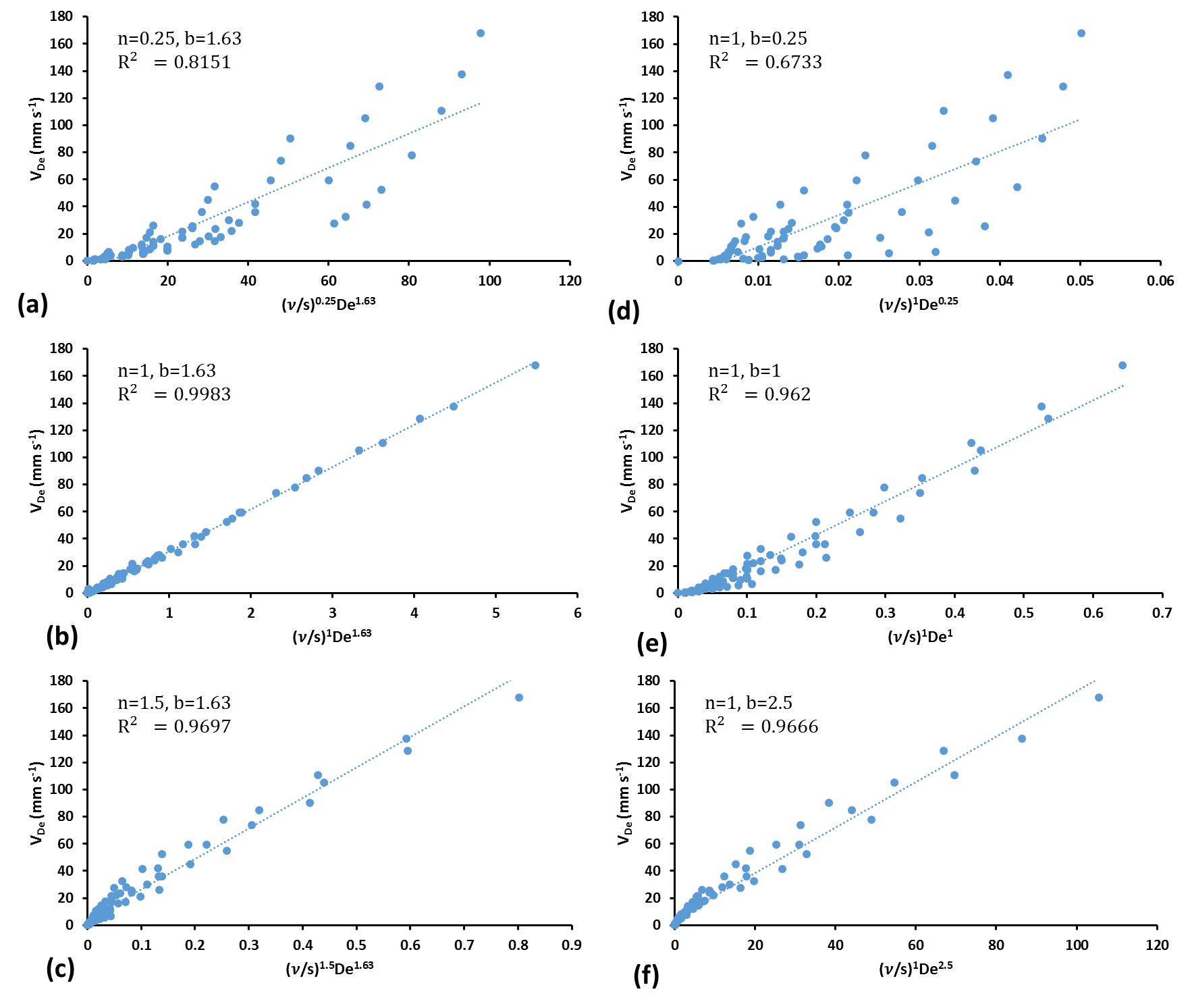


Fig. S2. Dean velocity plotted against (υ/s)^n^ De^b^ based on all numerical and experimental results obtained in this study. (a-c)The magnitude of b was set to 1.63 for similarity in De power to the commonly used value in the literature while n was varied from 0.25 to 1.5 to obtain the best fit (i.e. n=1). (d-f) n was set to 1 and b was varied from 0.25 to 2.5. Best fit was obtained when n=1 and b=1.63.


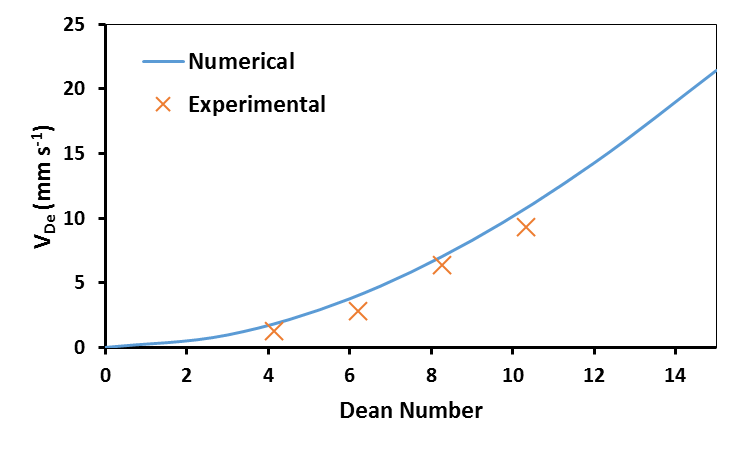


Fig. S3. Comparison between experimentally and numerically calculated Dean velocities (V_De_) in a device with R=1 cm and cross-sectional dimension of 100µm×150µm. Data points follow a power function and are in agreement with the numerical results.


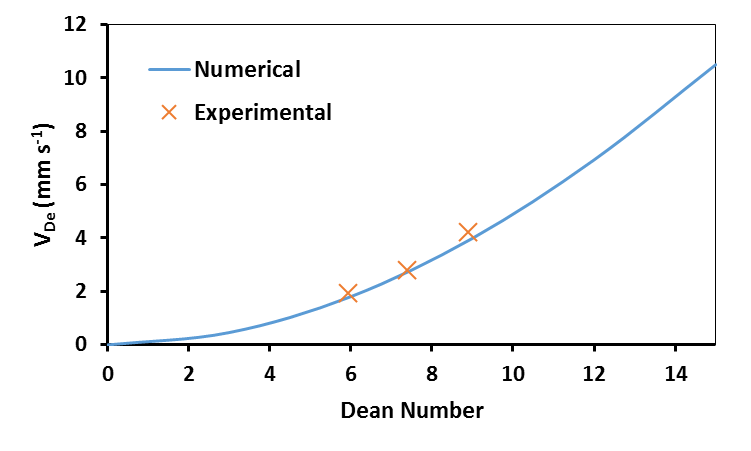


Fig. S4. Comparison between experimentally and numerically calculated Dean velocities (V_De_) in a device with R=1 cm and cross-sectional dimension of 300µm×150µm. Data points follow a power function and are in agreement with the numerical results.


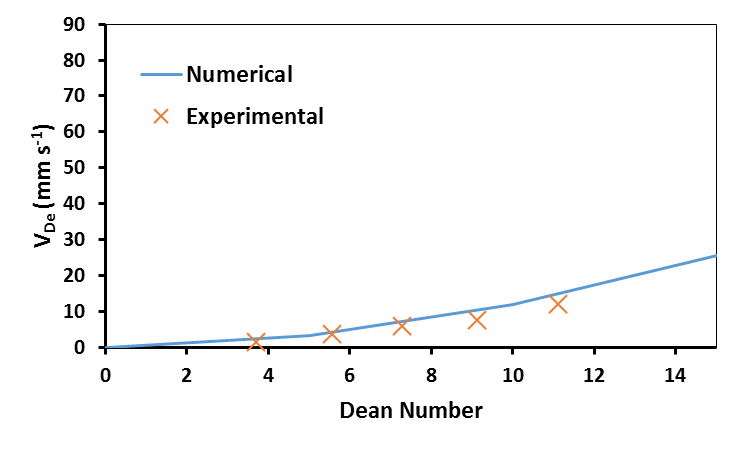


Fig. S5. Comparison between experimentally and numerically calculated Dean velocities (V_De_) in a device with R=0.5 cm and cross-sectional dimension of 150µm×150µm. Data points follow a power function and are in agreement with the numerical results.

**3. Supplementary Movies**

Movie S1. Switching of water and Methylene Blue (MB) in a curved microchannel with R=0.5 cm radius of curvature and cross-sectional dimensions of 150µm×150µm at Q=0.8 ml min^-1^. Water is introduced from the inner inlet while MB enters the channel from the outer inlet. The corresponding Dean number is De=10.9.

Movie S2. Water and MB switch their positions in a curved microchannel with R=1cm radius of curvature, cross section of 150µm×150µm at Q=1 ml min-1. (De= 9.6)

**Reference**

1. Cheng, N.-S. Formula for the Viscosity of a Glycerol−Water Mixture*. Ind. Eng. Chem. Re*s**. 4**7, 3285–3288 (2008).
